# Molecular surveillance of *pvdhfr*, *pvdhps*, and *pvmdr-1* mutations in *Plasmodium vivax* isolates from Yunnan and Anhui provinces of China

**DOI:** 10.1186/1475-2875-13-346

**Published:** 2014-09-02

**Authors:** Bo Huang, Shiguang Huang, Xin-zhuan Su, Xinxin Tong, Junping Yan, Hongbin Li, Fangli Lu

**Affiliations:** Department of Parasitology, Zhongshan School of Medicine, Sun Yat-sen University, Guangzhou, 510080 Guangdong China; Key Laboratory of Tropical Disease Control (Sun Yat-sen University), Ministry of Education, Guangzhou, 510080 Guangdong China; School of Medicine, Jinan University, Guangzhou, 510632 Guangdong China; Laboratory of Malaria and Vector Research, National Institute of Allergy and Infectious Diseases, National Institutes of Health, Bethesda, MD 20892 USA; State Key Laboratory of Cellular Stress Biology, School of Life Sciences, Xiamen University, Xiamen, 361005 Fujian China; Xishuangbanna CDC, Xishuangbanna Prefecture Jinghong, 666100 Yunnan China

**Keywords:** *Plasmodium vivax*, Polymorphism, Drug resistance markers, China

## Abstract

**Background:**

*Plasmodium vivax* is the predominant species of human malaria parasites present in China. Although sulphadoxine-pyrimethamine (SP) and chloroquine (CQ) have been widely used for malaria treatment in China, the resistance profiles of these drugs are not available. Analysis of dihydrofolate reductase (*dhfr*), dihydropteroate synthase (*dhps*), and multidrug resistance (*mdr-1*) gene mutations in *P. vivax* isolates is a valuable molecular approach for mapping resistance to SP and CQ. This study investigates the prevalence of *pvdhfr*, *pvdhps*, and *pvmdr-1* of *P. vivax* clinical isolates from China and provides baseline molecular epidemiologic data on SP- and CQ-associated resistance in *P. vivax*.

**Methods:**

*Plasmodium vivax* clinical isolates were collected from two malaria-endemic regions of China, subtropical (Xishuangbanna, Yunnan province) and temperate (Bozhou, Anhui province), from 2009 to 2012. All isolates were analysed for single nucleotide polymorphism haplotypes in *pvdhfr*, *pvdhps,* and *pvmdr-1* using direct DNA sequencing.

**Results:**

In *pvdhfr*, 15% of Xishuangbanna isolates carried wild-type (WT) allele, whereas the majority of isolates carried mutant genes with substitutions at five codons. Eight mutant haplotypes of *pvdhfr* were detected, while limited polymorphism of *pvdhfr* was found in Bozhou isolates. A size polymorphism was present in *pvdhfr,* with the three-repeat type being the most predominate in both Xishuangbanna (79%) and Bozhou (97%) isolates. In *pvdhps*, mutations at four codons were detected in Xishuangbanna isolates leading to six haplotypes, including WT allele, single-mutation, double-mutation, and triple-mutation alleles. All Bozhou isolates carried WT *pvhdps*. In *pvmdr-1*, isolates from Xishuangbanna carried mutations at codons Y976F and F1076L, whereas all isolates from Bozhou had only a single mutation at codon F1076L.

**Conclusions:**

*Plasmodium vivax* isolates from subtropical and temperate zones of China are shown to have dramatically different frequencies and patterns of mutations in *pvdhfr*, *pvdhps*, and *pvmdr-1.* Whereas *P. vivax* populations in subtropical China are highly resistant to SP and CQ, those in the temperate zone may still be susceptible to SP and CQ. This information is useful for establishing treatment policy and provides a baseline for molecular surveillance of drug-resistant *P. vivax* in these areas.

**Electronic supplementary material:**

The online version of this article (doi:10.1186/1475-2875-13-346) contains supplementary material, which is available to authorized users.

## Background

*Plasmodium vivax* infects approximately 100 million people annually and endangers 40% of the world’s population [1]. Malaria has been a significant public health problem in China until recently, predominantly in two areas of China: subtropical zone (Yunnan and Hainan provinces) and temperate zone (such as Anhui, Henan, Hunan, Hubei, and Jiangsu provinces). Due to China’s national malaria control programme launched in 1978, the intensity and scale of vivax malaria outbreaks have decreased dramatically through vector control and drug treatment of febrile individuals [2]; however, malaria incidence rose steadily between 2000 and 2006 [3]. In subtropical China, Yunnan is one of the two provinces with year-round local transmission of *P. vivax* and *Plasmodium falciparum* and has suffered one of the highest malaria morbidity and mortality rates in China. In temperate zone of Central China, outbreaks of malaria have been reported in Anhui and Henan provinces. In 2006, malaria prevalence increased considerably, with the highest numbers of malaria cases reported in Anhui province [4]. Although malaria incidence is low in China, the areas and populations at risk are large. *P. vivax* malaria accounted for > 95% of all malaria cases reported in China in 2007 [5], having become the sole parasite species and responsible for more than 90% of re-emerged malaria cases reported in temperate zone of China in 2009 [6]. In 2010, a countrywide malaria elimination policy was launched by the Ministry of Health of P. R. China with the goal of eliminating malaria by 2015 in China—with the exception of the border region in Yunnan province, and to completely eliminate malaria from China by 2020. Therefore, there is an urgent need to monitor vivax malaria transmission and drug resistance in subtropical and temperate zones of China.

Resistance to common anti-malarial drugs, such as chloroquine (CQ), has been reported for *P. vivax* around the world, including Indonesia [7], Myanmar [8], India [9], Vietnam [10], Turkey [11], and Ethiopia [12]. CQ and primaquine have been used as first-line therapies for radical cure of vivax malaria in China for the past 60 years, and clinical failures or reduced efficacy of CQ-primaquine drug combinations for treating vivax malaria had been reported at the Yunnan-Myanmar border [13], in Yunnan [14] and Anhui [15] provinces. Although sulphadoxine-pyrimethamine (SP) is rarely used to treat patients with *P. vivax* malaria, pyrimethamine, a component of the two combination regimens [Maloprim® (plus dapsone) and Fansidar® (plus sulphadoxine)], has been widely used for malaria prophylaxis in China between the mid-1960s and early 1990s [16]. This may place *P. vivax* under the selection of SP drug stress in China, especially in the subtropical zone where *P. vivax* and *P. falciparum* mixed-species infections were common. *P. vivax* malaria re-emerged in many counties of China in recent years, possibly caused by social and environmental changes and intrinsic differences in parasites with long or short relapse patterns [17–20]. Even changes in mosquito strains or species could favour a subpopulation of vivax parasites and lead to changes in the parasite population structure [21], and the emergence and spread of drug-resistant malaria should, of course, be held accountable. Studies in other *P. vivax*-endemic regions such as Vietnam highlight the geographic heterogeneity and temporal dynamics of drug resistance in *P. vivax* [10], raising further concerns about the presence of clinical resistance to SP and CQ in these areas of China and highlighting the importance of continued surveillance of CQ drug efficacy.

Molecular genetic markers of resistance are useful for monitoring the emergence and spread of anti-malarial drug resistance; a better understanding of the mechanisms of drug action and resistance are essential for fulfilling the promise of eradicating malaria [22]. Currently, several genetic markers, including dihydrofolate reductase (*dhfr*), dihydropteroate synthase (*dhps*), and multidrug resistance (*mdr-1*), have been used to study the prevalence and spread of SP- and CQ-resistant *P. vivax* [23–26]; however, molecular epidemiologic information on *P. vivax* parasite resistance to CQ and primaquine in China is limited. This study investigated the frequencies and patterns of mutations in *pvdhfr*, *pvdhps*, and *pvmdr-1* linked to SP and CQ resistance in *P. vivax* isolates from Yunnan and Anhui provinces of China, and the results provide important information for molecular surveillance of drug-resistant *P. vivax* in these areas.

## Methods

### Study areas

**Figure 1:**
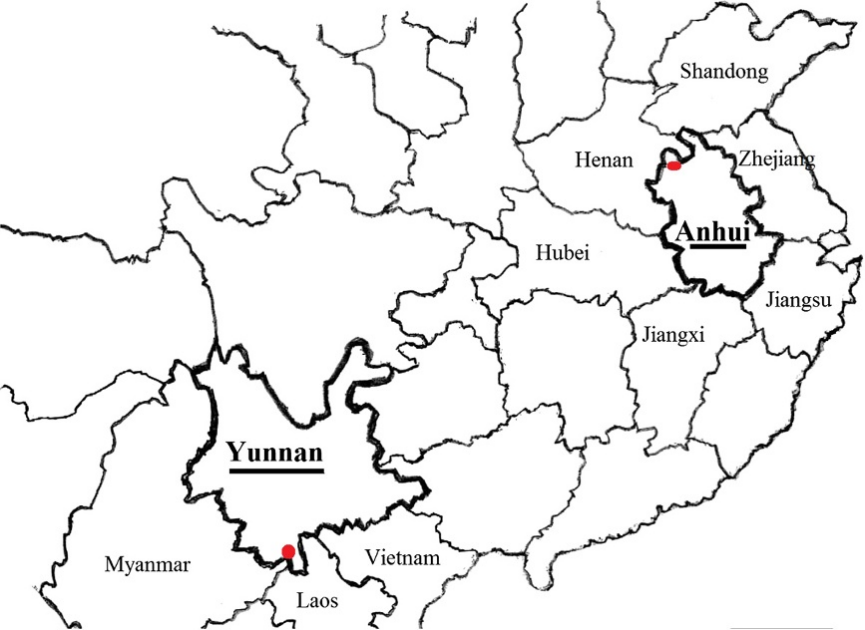
**Map of Yunnan in subtropical zone and Anhui province in temperate zone of China.** Shown are the locations of Xishuangbanna prefecture and Bozhou city where *P. vivax* isolates were collected. Scale bar = 400 kilometers.

The study was conducted in areas in subtropical (Xishuangbanna prefecture, Yunnan province) and temperate (Bozhou city, Anhui province) zones of China (Figure 1). Xishuangbanna prefecture had a population of around 113,000 in 2010. It is located in southern Yunnan (the south-west area of China), bordering Myanmar in the west and Laos and Vietnam in the south, in an area between 21°08’ and 22°36’ latitude and 99°56’ and 101°50’ longitude. Its climate is characterized by a cool, dry winter, and a warm, wet summer. It has a distinct seasonal climate characterized by rainy season from May to October and dry season from November to May, an average temperature of 18–22°C and average rainfall of 1100–2400 mm per year.

Xishuangbanna was a highly endemic area for both *P. falciparum* and *P. vivax* malaria. Five confirmed *Anopheles* species are widely distributed throughout Yunnan province, with the predominant malaria vectors being *Anopheles minimus* and *Anopheles dirus* [27]. Transmission in the study area is seasonal, and malaria incidence characterized by peaks of infection occurring from June to July and again in October to November [18]. Bozhou city’s population was around 485,000 in 2010. It is located in the north-west of Anhui (temperate zone of China) bordering six provinces: Jiangsu in the south-east, Shandong in the north, Hubei in the west, Henan in north-west, Jiangxi in the south, and Zhejiang in the north-east, located in an area between 32°51’ and 35°05’ latitude and 115°53’ and 116°49’ longitude. The area has a distinct seasonal climate characterized by humid summer from June to September and dry winter from October to December, with an average temperature of 14–17°C and average rainfall of 500–750 mm per year [16]. Bozhou city was an endemic area for *P. vivax* malaria, and the main malaria vector was *Anopheles sinensis* and the secondary vector *Anopheles anthropophagus*.

### Blood sample collection

Blood samples were collected from patients with symptomatic *P. vivax* malaria admitted to local hospitals for anti-malarial drug treatment. Inclusion criterion was patients infected with *P. vivax* but no other human malaria species as confirmed by peripheral smear examination. Preparation and staining of blood slides were done following the World Health Organization procedures for basic malaria microscopy. Thick and thin blood smears were checked after staining with Giemsa. A total of 114 *P. vivax* clinical isolates (blood samples) were collected from patients with symptomatic *P. vivax* malaria admitted to local hospitals for anti-malarial drug treatment (n = 53) in Xishuangbanna, Yunnan province, from June 2011 to October 2012 and (n = 61) in Bozhou city, Anhui province, from June 2009 to October 2010 (collected earlier than those of Xishuangbanna samples). Two patients from Bozhou city had a travel history outside of Anhui; all others had no history of travel to neighbouring provinces or countries. All the Xishuangbanna isolates were collected from the border of Myanmar. After informed consent from all adults or legal guardians of children, a 1.0 mL sample of whole blood was collected from vivax malaria patients in EDTA tubes and stored at -20°C until DNA extraction. This study was reviewed and approved by the Ethics Committee of Sun Yat-sen University, China (IRB 2009A0101).

### DNA extraction for *pvdhfr*, *pvdhps*, and *pvmdr-1*

*Plasmodium vivax* genomic DNA was extracted from 200 μL of each blood sample using a DNA blood kit following the manufacturer’s instructions (Takara Bio Inc., Shiga, Japan) with minor modifications. The DNA was dissolved in TE buffer (10 mM Tris–HCl, 0.1 M EDTA, pH 8.0) and stored at -20°C until use. The quality of total DNA was analysed by running 5 μL of each DNA sample on a 1.0% agarose gel stained with ethidium bromide and visualized under ultraviolet illumination.

### Polymerase chain reaction (PCR) amplification for *pvdhfr*, *pvdhps*, and *pvmdr-1*

To amplify *pvdhfr*, *pvdhps*, and *pvmdr-1*, a nested PCR amplification method was used following previously reported protocols with some minor modifications [15, 16, 28]. Oligonucleotide primers and cycling conditions are listed in Table 1. All amplification reactions were carried out in a total volume of 25 μL containing 17.0 μL of dH_2_O, 1.0 μL of each primer (10 pM), 1.5 μL of MgCl_2_ (25 mM), 0.5 μL of Taq polymerase (5 U/μL), 0.5 μL of dNTP mixture (10 mM each), and 2.5 μL of 10 × PCR buffer following the manufacturer’s instructions (BioBasic Inc., Markham, ON, Canada). Primary amplification reactions were initiated with 1.0 μL of template genomic DNA prepared from the blood samples, and 1.0 μL of primary reaction amplification was used in secondary amplification reactions. The amplified nested PCR products were resolved on 1.0% agarose gel, and the sizes of the PCR products were determined using a 100-bp DNA ladder (NewProbe, Beijing, China). The nested PCR products were stored at -20°C until analysis.

**Table 1:** **Sequences of primers and cycling conditions used to amplify** ***pvdhfr*** **,** ***pvdhps*** **, and** ***pvmdr-1 of Plasmodium vivax isolates*** ^a,b^

Genes ^c^	Primers ^d^	PCR cycling conditions ^e^	Product size (bp)	Reference
*pvdhfr* (P)	F: 5’- CACCGCACCAGTTGATTCCT-3’	95°C 5 min/[95°C 30 s, 58°C 30 s, 68°C 1 min] × 30 cycles, 68°C 7 min	979	[16]
	R: 5’- CCTCGGCGTTGTTCTTCT-3’			
*pvdhfr* (S)	F: 5’- CCCCACCAC ATA ACG AAG-3’	95°C 5 min/[95°C 30 s, 58°C 30 s, 68°C 45 s] × 30 cycles, 68°C 7 min	755	[16]
	R: 5’- CCC CACCTT GCTGTA AACC-3’			
*pvdhps* (P)	F: 5’-GATGGCGGTTTATTTGTCG-3’	95°C 5 min/[95°C 30 s, 59°C 30 s, 68°C 1 min] × 30 cycles, 68°C 7 min	1009	[16]
	R: 5’-GCTGATCTTTGTCTTGACG-3’			
*pvdhps* (S)	F: 5’-GCTGTGGAGAGGATGTTC-3’	95°C 5 min/[94°C 30 s, 59°C 30 s, 68°C 45 s] × 30 cycles, 68°C 7 min	731	[16]
	R: 5’-CCGCTCATCAGTCTGCAC-3’			
*pvmdr-1* (P)	F: 5’-ACGACATGATCCAAACGACA-3’	94°C 2 min/[94°C 30 s, 60°C 30 s, 72°C 3 min] × 35 cycles, 72°C 5 min	2784	[28]
	R: 5’-CTTATATACGCCGTCCTGCAC-3’			
*pvmdr-1* (S)	F: 5’-GGATAGTCATGCCCCAGGATTG-3’	94°C 10 min/[94°C 40 s, 62°C 1 min, 72°C 1 min] × 35 cycles, 72°C 10 min	604	[15]
	R: 5’-CATCAACTTCCCGGCGTAGC-3’			

^a^ *P. vivax* isolates from Yunnan and Anhui provinces.

^b^The data provided oligonucleotide primers and cycling conditions of *pvdhfr*, *pvdhps*, and *pvmdr-1* genes.

^c^P, Primary PCR reaction; S, Secondary PCR reaction.

^d^F, Forward primer; R, Reverse primer.

^e^Cycling conditions were modified in the present study.

### Sequence analysis of *pvdhfr*, *pvdhps*, and *pvmdr-1*

The nested PCR products of *pvdhfr*, *pvdhps*, and *pvmdr-1* from the above samples were directly sequenced in both directions, using an ABI PRISM3730 DNA sequencer from Sangon Biotech (Shanghai, China). Nucleotide and amino acid sequences of *pvdhfr*, *pvdhps*, and *pvmdr-1* were aligned and compared with the following published sequences of *pvdhfr* (accession no. X98123), *pvdhps* (accession no. AY186730), and *pvmdr-1* (accession no. AY618622) using Clustal W of the BioEdit 7.0 program.

### Statistical analysis

Statistical significance was determined with SPSS software (version 13.0). U test was used to compare the prevalence of infection between collection sites. *P* < 0.05 was considered statistically significant.

## Results

### Prevalence and patterns of mutant *pvdhfr*in *P. vivax*isolates

All blood samples (n = 114) were shown to be infected with *P. vivax* only by microscopy. The *pvdhfr* gene was successfully amplified from all 114 *P. vivax* isolates. Mutations at codons F57I/L, S58R, T61M, H99R/S, and S117N/T were detected in the 53 Xishuangbanna isolates, accounting for 60%, 62%, 62%, 19%, and 74% of the isolates examined, respectively (Table 2); no mutations at *pvdhfr* positions I13 or I173 were found in the Xishuangbanna isolates. Only H99S and S117N were detected in the 61 Bozhou isolates, with frequencies of 40% and 57% isolates, respectively. Mutant codon S117N was the most prevalent in both Xishuangbanna and Bozhou areas, accounting for 74% and 57% of isolates examined, respectively. Haplotype analysis of *pvdhfr* for all Xishuangbanna isolates revealed eight distinct allelic forms (Table 3), including the wild-type (WT) allele (I13/F57/S58/T61/H99/S117/I173), two single-mutant alleles (99S and 117N), three double-mutant alleles (99S/117N, 99R/117N, and 58R/99N), and two quadruple mutant alleles (57I/58R/61M/117T and 57L/58R/61M/117T). Of the eight allelic variants, the most prevalent allelic variants were quadruple-mutant alleles (57L/58R/61M/117T) (53%, 28/53) among Xishuangbanna isolates, followed by WT allele (15%, 8/53) and a single-mutant allele H99S (11%, 6/53). The remaining five allelic variants were only detected in 21% (11/53) of *P. vivax* isolates examined from Xishuangbanna. Four allelic variants [WT allele, single-mutant alleles (99S and 117N), and a double-mutant allele (99S/117N)] were detected in 61 isolates from Bozhou, with a single-mutant allele (117N) being predominant (42%, 26/61) (Table 3).

**Table 2:** **Prevalence of mutations conferring resistance to chloroquine and sulfadoxine-pyrimethemine in** ***Plasmodium vivax*** **isolates** ^a^

Gene locus		Number of isolates (% ^b^)		
		Subtropical zone,	Temperate zone,	Total,
		***n*** = 53	***n*** = 61	***n*** = 114
*dhfr*	Mutation at codon 13	0 (0)	0 (0)	0 (0)
	Mutation at codon 57	32 (60**)	0 (0)	32 (28)
	Mutation at codon 58	33 (62**)	0 (0)	33 (29)
	Mutation at codon 61	33 (62**)	0 (0)	33 (29)
	Mutation at codon 99	10 (19**)	24 (40)	34 (30)
	Mutation at codon 117	39 (74)	35 (57)	74 (65)
	Mutation at codon 173	0 (0)	0 (0)	0 (0)
*dhps*	Mutation at codon 382	17 (32**)	0 (0)	17 (15)
	Mutation at codon 383	42 (79**)	0 (0)	42 (37)
	Mutation at codon 512	1 (2)	0 (0)	1 (1)
	Mutation at codon 553	15 (28**)	0 (0)	15 (13)
	Mutation at codon 585	0 (0)	0 (0)	0 (0)
*mdr-1*	Mutation at codon 976	5 (9**)	0 (0)	5 (4)
	Mutation at codon 1076	19 (36**)	61 (100)	80 (70)

^a^Isolates from subtropical (Yunnan province) and temperate (Anhui province) zones of China.

^b^Statistically significant differences for comparison with isolates from temperate zone (Anhui province) of China (**P < 0.01) using *u*-test.

**Table 3:** **Prevalence of single nucleotide polymorphisms and multi-mutated haplotypes in** ***Plasmodium vivax dhfr*** **,** ***dhps*** **, and** ***mdr-1*** ^**a**^

			Number of isolates (% ^b^)		
Genotype			Subtropical zone,	Temperate zone,	Total,
			***n*** = 53	***n*** = 61	***n*** = 114
*dhfr*	Wild-type haplotype	I_13_F_57_S_58_T_61_H_99_S_117_I_173_	8 (15)	11 (18)	19 (17)
	Single-mutant haplotype	I_13_F_57_S_58_T_61_ **S**_99_S_117_I_173_	6 (11)	15 (24)	21 (18)
	Single-mutant haplotype	I_13_F_57_S_58_T_61_H_99_ **N**_117_I_173_	2 (4**)	26 (42)	28 (25)
	Double-mutant haplotype	I_13_F_57_S_58_T_61_ **S**_99_ **N**_117_I_173_	1 (2**)	9 (15)	10 (9)
	Double-mutant haplotype	I_13_F_57_S_58_T_61_ **R**_99_ **N**_117_I_173_	3 (6*)	0 (0)	3 (3)
	Double-mutant haplotype	I_13_F_57_ **R**_58_T_61_H_99_ **N**_117_I_173_	1 (2)	0 (0)	1 (1)
	Quadruple-mutant haplotype	I_13_ **L**_57_ **R**_58_ **M**_61_H_99_ **T**_117_I_173_	28 (53**)	0 (0)	28 (25)
	Quadruple-mutant haplotype	I_13_ **I**_57_ **R**_58_ **M**_61_H_99_ **T**_117_I_173_	4 (8*)	0 (0)	4 (4)
*dhps*	Wild-type haplotype	S_382_A_383_K_512_A_553_V_585_	11 (21**)	61 (100)	72 (64)
	Single-mutant haplotype	S_382_ **G**_383_K_512_A_553_V_585_	13 (25**)	0 (0)	13 (11)
	Double-mutant haplotype	**A**_382_ **G**_383_K_512_A_553_V_585_	14 (26**)	0 (0)	14 (12)
	Double-mutant haplotype	S_382_ **G**_383_K_512_ **G**_553_V_585_	11 (21**)	0 (0)	11 (10)
	Triple-mutant haplotype	**A**_382_ **G**_383_K_512_ **G**_553_V_585_	3 (6*)	0 (0)	3 (3)
	Triple-mutant haplotype	S_382_ **G**_383_ **E**_512_ **G**_553_V_585_	1 (2)	0 (0)	1 (1)
*mdr-1*	Wild-type haplotype	Y_976_F_1076_	34 (65**)	0 (0)	34 (30)
	Single-mutant haplotype	Y_976_ **L**_1076_	14 (26**)	61 (100)	75 (66)
	Double-mutant haplotype	**F**_976_ **L**_1076_	5 (10**)	0 (0)	5 (4)

^a^In isolates from subtropical (Yunnan province) and temperate (Anhui province) zones of China. The mutated amino acids are indicated by bold type.

^b^Statistically significant differences for comparison with isolates from temperate zone (Anhui province) of China (*, *P* < 0.05; **, *P* < 0.01) using *u*-test.

**Figure 2:**
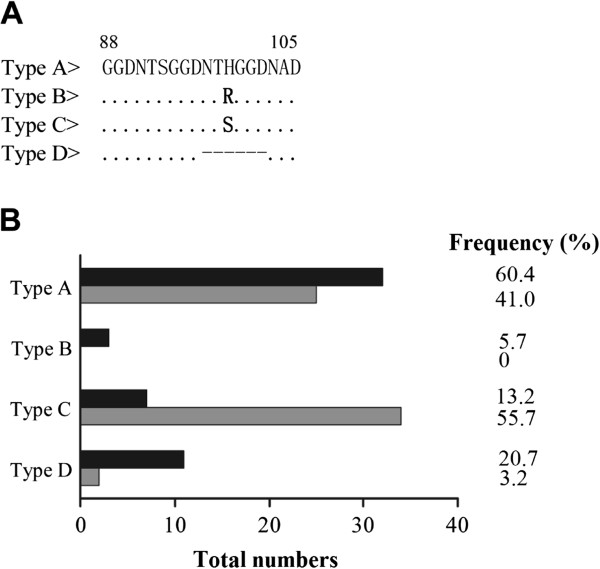
**Alignment of central tandem repeat region between amino acid positions 88 and 105 in the** ***pvdhfr*** **gene and frequencies of central tandem repeat types circulating from Xishuangbanna and Bozhou. A)** Sequences were constructed to the published amino acid sequences (accession no. X98123). Dots (. . . .) and dashes (---) represent identical residues and deletions, respectively. Amino acid changes resulting from nucleotide substitutions are shown in bold. **B)** Frequencies of four central tandem repeat types obtained from 53 *P. vivax* Xishuangbanna isolates (black) and 61 Bozhou isolates (grey).

In addition to the above point mutations, variations at the central tandem repeat region between amino acid positions 88 and 105 of *pvdhfr* were also detected. Analysis of repeat variant in *pvdhfr* by sequencing showed that most (89%, 101/114) of the isolates from both areas contained three copies of the GGDNXX repeat that could be divided into type A, B, and C based on substitutions in the middle repeat (Figure 2A). The rest of the isolates (11%, 13/114) had two repeats (type D). The majority of parasites from both Xishuangbanna and Bozhou isolates (79% and 97%, respectively) had the three-copy repeat type. However, the distribution and frequency of tandem repeats in *pvdhfr* in Xishuangbanna isolates were different from those of Bozhou isolates (Figure 2B). Type A (60%) was the dominant allele in Xishuangbanna, whereas type C (56%) was the most common allele among the Bozhou isolates.

### Prevalence and patterns of mutant *pvdhps*in *P. vivax*isolates

All 114 isolates from Xishuangbanna and Bozhou were successfully amplified for *pvdhps*. Sequence analysis revealed that point mutations were detected at codons S382A, A383G, K512E, and A553G, but not at V585 in *pvdhps* in Xishuangbanna isolates (Table 2). Among the above four mutants, codon S382A was the most prevalent, accounting for 79%, followed by codons S382A and A553G with frequency of 32% and 28% in all Xishuangbanna isolates, respectively; the rest of the mutant codon K512E accounted for only 2% of the Xishuangbanna isolates. Haplotype analysis of *pvdhps* in Xishuangbanna isolates showed six distinct allelic forms, including WT allele (S382/A383/K512/A553/V585), one single-mutant allele (383G), two double-mutant alleles (S382A/A383G and 383G/553G), and two triple-mutant alleles (S382A/A383G/A553G and 383G/512E/585G) (Table 3). The most common *pvdhps* alleles in Xishuangbanna isolates were the double-mutant allele 382A/383G, followed by single-mutant allele 383G, double-mutant allele 383G/553G, and WT mutant, accounting for 26%, 25%, 21%, and 21%, respectively. However, no mutation was detected in any of the Bozhou *P. vivax* isolates (Tables 2 and 3).

### Prevalence and patterns of mutant *pvmdr-1*in *P. vivax*isolates

All 114 samples were successfully amplified for *pvmdr-1*, and mutations at codons Y976F and F1076L with the frequencies of 4% and 70%, respectively, were detected (Table 2); however, different mutant alleles were found in the parasite populations from Xishuangbanna and Bozhou. Mutant codon F1076L was detected from the isolates of both Xishuangbanna and Bozhou (36% and 100%, respectively); whereas mutant codon Y976F was found only in Xishuangbanna isolates (9%). Three haplotypes existed among the parasites: WT (Y976/ F1076), single-mutant allele (1076L), and double-mutant allele (976F/1076L), with WT being the predominant type (65%, 34/53) in Xishuangbanna isolates (Table 3) and the single-mutant allele (1076L) being the only genotype (100%, 61/61) in the Bozhou isolates (Table 3).

### Haplotypes of combined *pvdhfr*, *pvdhps*, and *pvmdr-1*allelic variants

Analysing variants of the three anti-malarial drug genetic markers (*pvdhfr*, *pvdhps*, and *pvmdr-1*) in combination identified a total of 28 distinct haplotypes in the 114 isolates (24.5%) from both Xishuangbanna and Bozhou. Among the haplotypes, 26 were found in Xishuangbanna isolates (22.8%), four were found in Bozhou isolates (3.5%), and two (Types 2 and 10) were found in both regions (Additional file 1). The two most prevalent haplotypes were Types 21 and 22, accounting for 11.3% and 13.2% of Xishuangbanna isolates, respectively; the remainder haplotypes were distributed at low frequency (1.9% to 7.5%), although the predominant haplotype in Bozhou isolates was Type 7 (42.6%, 26/61), followed by Types 10, 2, and 13. Disregarding the mutations at codon 99 in *pvdhfr* and at codon 1076 in *pvmdr-1*, which were considered to be not related with the anti-malarial drugs, 4.4% of the 114 isolates (including Types 16, 19, and 26) had mutations in all the three genes, *pvdhfr*, *pvdhps*, and *pvmdr-1,* simultaneously, whereas 31.6% of the 114 isolates (including Types 8, 14–19, and 21–28) had mutations in the SP drug-related genes (*dhfr* and *dhps*) which were found in Xishuangbanna but not Bozhou isolates.

## Discussion

Emergence and spread of CQ- or SP-resistant *P. vivax* strains has been reported in many malarial regions around the world, especially in Southeast Asia, which is responsible for the increasing morbidity and mortality of *P. vivax* [29]. The use of anti-malarial drugs exhibits enormous geographic heterogeneity in subtropical and temperate zones of China. With increasing movement of human populations, drug-resistant *P. vivax* populations and the parasite transmission pattern in the regions are also changing constantly. The emergence and spread of drug-resistant strains of human *Plasmodium* species may be partly responsible for re-emergence of malaria in China. SP was widely used for malaria prophylaxis between the mid-1960s and early 1990s, and CQ-primaquine is still the first-line anti-malarial drug for treating *P. vivax* malaria in China [16]. Molecular markers have been validated as tools for surveillance of resistance. Analysis of point mutations in these marker genes thus serves as a valuable molecular approach for mapping drug resistance and monitoring malaria-control measures [30]. In this study, the profile of mutations in marker genes associated with CQ and antifolate drug resistance among the *P. vivax* parasites obtained from patients of the subtropical (Xishuangbanna in Yunnan) and temperate (Bozhou in Anhui) zones of China were determined to better understand the current and changing patterns of CQ- and SP-resistant *P. vivax* in different malaria-endemic areas. The *P. vivax* parasite populations in subtropical and temperate zones were shown to differ dramatically in *pvdhfr*, *pvdhps*, and *pvmdr-1* allele frequencies, i.e., *P. vivax* populations in subtropical zone are mostly resistant to SP and are likely more tolerant to CQ, whereas the majority of *P. vivax* populations in the temperate zone are still effectively susceptible to SP and CQ.

The long history of CQ use has exposed *P. vivax* to this drug pressure continually in China. Clinical failures after standard CQ treatment were reported in four cases of *P. vivax* malaria in Yunnan province in 1996 [14]. A trend of gradual decline of *in vitro* sensitivity to CQ had also been documented between 2005 and 2008 in some areas of China, especially at the Yunnan-Myanmar border and in central China [31], and reduced efficacy of CQ combinations for treating vivax malaria patients from 2007 to 2008 was reported at the Yunnan-Myanmar border [13]. The relatively high frequency (9%) of *pvmdr-1* Y976F mutation among the Xishuangbanna isolates is consistent with the previous reports of declining sensitivity to CQ. The Y976F mutation had been reported in *P. vivax* isolates from many malaria-endemic regions around the world, including Indonesia [26], Thailand [32], Myanmar [32], and Mauritania [33, and is associated with an increase in CQ IC_50_ values *in vitro* [15]. Cases of CQ-resistant *P. vivax* malaria have been reported in the areas of Yunnan bordering Myanmar, Laos, and Vietnam [8, 10]. The long history of CQ use, as well as frequent population movement across the borders, may contribute to the CQ-resistant *P. vivax* detected in Xishuangbanna. However, the Y976F mutation was not detected in any Bozhou isolates, which is consistent with the results showing that CQ is still effective in killing *P. vivax* parasites from the temperate zone in an *in vitro* schizont maturation inhibition assay [15]. The presence of Y976F in *pvmdr-1* in Xishuangbanna isolates suggests a trend toward decreased CQ sensitivity.

This study detected the F1076L mutation in *pvmdr-1* in 100% of Bozhou isolates, which is consistent with previous reports from Central China (100%) [26], the Republic of Korea (100%) [26], Mauritania (100%) [26], and Madagascar (100%) [34]; however, only 36% of Xishuangbanna isolates had the F1076L mutation. It has been reported that the F1076L substitution may not be linked to drug resistance but may rather be a geographic variant [34].

SP inhibits the activities of *dhps* and *dhfr* in the folate biosynthesis pathway of both *P. falciparum* and *P. vivax* parasites, resulting in a synergistic anti-malarial effect [35]. Resistance to antifolate drugs in both parasite species is found to be involved with point mutations in *dhps* and *dhfr* [23]. Although *P. vivax* infections are not generally treated with SP, the parasite is often exposed to the drug due to mixed infections and misdiagnosis [36]. In this study, five mutations at amino acid positions 57, 58, 61, 99, and 117 and seven mutated alleles in *pvdhfr* were detected in Xishuangbanna isolates, while only mutations at positions 99 and 117 of *pvdhfr* and three mutated alleles in *pvdhfr* were found in Bozhou isolates. It has been reported that double mutation S58R and S117N in *pvdhfr* may first arise under drug pressure and move toward development of resistance to SP [23]. Interestingly, approximately 62% of Xishuangbanna isolates was found to have double mutations at S58R/S117N, whereas no Bozhou isolates were detected with these mutations. Instead, 39% of Bozhou isolates had a single S117N mutation, indicating that the *P. vivax* isolate circulating in Xishuangbanna may be under stronger drug pressure than those in Bozhou. The most prevalent double mutations of S58R/S117N in Xishuangbanna isolates were similar to those in previous reports from East Timor [37], the Philippines [37], Vietnam [37], and Pakistan [38]. Clinical studies have shown that patients carrying triple and quadruple *pvdhfr* mutant vivax parasites were more likely associated with SP treatment failure than those with WT parasites [39, 40]. The triple and quadruple mutations in *pvdhfr* have been detected in Yunnan [16], Hainan [16], and Guizhou [41] provinces of China’s subtropical zone, and in some other countries including Indonesia [40], Papua New Guinea [40] and India [42]. Similarly, 61% of Xishuangbanna isolates had quadruple mutant alleles (57L/58R/61M/117T and 57I/58R/61M/117T), strongly suggesting that many Xishuangbanna *P. vivax* isolates are resistant to sulphadoxine drugs. The triple and quadruple mutations were not detected in isolates in Bozhou as well as in other temperate areas of China (such as Wuhe county in Anhui province) [41], Thailand [43], Vietnam [43], Korea [43], Afghanistan [44], Pakistan [45], and Iran [46], indicating that Bozhou *P. vivax* isolates may still be sensitive to antifolate drugs.

Among mutated codons in *pvdhfr*, the T61M mutation was mostly linked to the S117T mutation [23]. In the present study, two mutations at T61M/S117T with quadruple mutated alleles (57L/58R/61M/117T and 57I/58R/61M/117T) were detected in Xishuangbanna isolates. The observation is consistent with the early reports from Thailand [47], but is in contrast to other reports from the temperate zone of China (Wuhe county in Anhui province) that most T61M mutations were found as single mutations and arose independently [41]. In the current study, the three-copy repeat type was the predominate type in both Xishuangbanna (79%) and Bozhou (97%) isolates, which was similar to the findings from India [42], Afghanistan [44], Iran [46], and Wuhe county in Anhui province and Luodian county in Guizhou province of China [41]. The association between *pvdhfr* mutations and tandem repeat polymorphisms has been extensively investigated and used to predict the prevalence of drug-resistant malaria around the world [43]. Therefore, the observations from this study suggest increasing prevalence of antifolate-resistant parasites in the Xishuangbanna region. Additionally, no isolates were detected with the four-copy repeat type from either Xishuangbanna or Bozhou, in contrast to other reports showing that quadruple-mutant *dhfr* alleles were exclusively associated with the four-copy repeat type in Thailand [23], India [42], and Myanmar [48]. Whether the four-copy repeat type contributes to drug resistance requires further investigation.

It has been suggested that mutations at codons 382, 383, 512, 553, and 585 in *pvdhps* gene are related to reduced sensitivity to sulfadoxine [46, 49]. In the present study, four *pvdhps* mutations at codons S382A (32%), A383G (79%), K512E (2%), and A553G (28%) and five mutant-allelic types were detected in Xishuangbanna isolates, including 25% single-mutant allele (A383G), 47% double-mutant allele (S382A/A383G and A383G/A553G), and 8% triple-mutant allele (S382A/A383G/A553G and A383G/K512E/A553G), strongly suggesting that Xishuangbanna *P. vivax* isolates were highly resistant to sulphadoxine. The high prevalence of mutant *pfdhps* alleles is similar to a previous report in Yunnan province of China (Nu River) [16]. In contrast, all isolates from Bozhou carried the WT allele, which is similar to reports from Wuhe county in Anhui province [31, 41] and Luodian county in Guizhou province [41] of China, Mauritania [33], Iran [38], and Afghanistan [44]. The absence of mutations in *pvdhps* in this study indicated that *P. vivax* populations in Bozhou may still be effectively susceptible to sulphadoxine.

Molecular epidemiologic studies in different areas have shown dramatically different mutation rates in *pvdhfr* and *pvdhps*, which may be attributed to different drug-selection pressures or the intrinsic differences among endemic strains of *P. vivax*. It is important to take into account the presence of A383G mutation of *pvdhps* along with double *pvdhfr* mutations, as SP drug-treatment failure was more frequently associated with multiple mutations in *pvdhfr* and *pvdhps* [50]; when the *P. falciparum* parasite carries mutant alleles of *dhfr* and *dhps*, clinical effectiveness of SP is compromised [51]. In the current study, disregarding the mutations at codon 99 of *pvdhfr*, which is not considered to be related to the anti-malarial drugs, 31.6% of 114 isolates (including Types 8, 14 to 19, and 21 to 28) contained the mutations in *pvdhfr* and *pvdhps* and 4.4% of 114 isolates (including Types 16, 19, and 26) mutated simultaneously in the three drug-resistant genes *pvdhfr*, *pvdhps*, and *pvmdr-1* were found in the Xishuangbanna isolates but not in Bozhou isolates. Although SP was not recommended to treat *P. vivax* malaria in China due to the intensive use of antifolate drugs for treating *P. falciparum* infections, *P. vivax* has been under SP drugs pressure through mixed infections and/or incorrect diagnosis in Yunnan. In contrast, without the introduction of sulphadoxine in Anhui—where *P. vivax* is the only or most predominant malaria parasite, *P. vivax* is not under sulphadoxine but rather pyrimethamine drug pressure. Therefore, the different usage of antifolate drug in Yunnan and Anhui may lead to the high prevalence of *pvdhfr* and *pvdhps* mutated alleles in Xishuangbanna isolates and relatively low prevalence of these mutations in Bozhou isolates. In tropical area, in addition to longer transmission period, short-term relapses are more common (1–2 months), leading to increased expose of *P. vivax* parasites to sub-therapeutic concentrations of SP and CQ. Regardless, the results from this study indicates that the high prevalence of multiple mutations of *pvdhfr*, *pvdhps*, and *pvmdr-1* genes may further reduce the sensitivity to SP and CQ in *P. vivax* populations in Xishuangbanna of Yunnan, while the *P. vivax* population may still be susceptible to SP and CQ in Bozhou of central China due to the absence of multiple mutations in *pvdhfr*, *pvdhps*, and *pvmdr-1*.

## Conclusions

Results from the current study indicate that the prevalence and patterns of mutant *pvdhfr*, *pvdhps*, and *pvmdr-1* exhibit enormous geographic heterogeneity in subtropical (Xishuangbanna, Yunnan) and temperate (Bozhou, Anhui) zones of China. *P. vivax* populations in subtropical zone of China are likely resistant to SP and CQ, whereas in temperate zone of China they are still relatively susceptible to SP and CQ, which provides valuable information for monitoring drug resistance in different malaria-endemic areas of China and for assessing the appropriateness of the current national anti-malarial drug policy. The increased resolution afforded by combination of molecular genetics and geographic information systems (GIS) tools has the potential to provide insights into the epidemiology, evolution, and ecology of these parasites in the future.

## Electronic supplementary material

Additional file 1: **Frequency distribution of SNPs in combination of** ***pvdhfr*** **,** ***pvdhps*** **, and** ***pvmdr-1*** **haplotypes associated with SP and CQ in** ***P. vivax*** **isolates.** Description: The data provided frequency distribution of SNPs in combination of *pvdhfr*, *pvdhps*, and *pvmdr-1* haplotypes. (DOCX 34 KB)

## Abbreviations

CQ: Chloroquine
*dhfr*: Dihydrofolate reductase gene
*dhps*: Dihydropteroate synthase gene
*mdr-1*: Multidrug resistance gene
PCR: Polymerase chain reaction
SP: Sulphadoxine-pyrimethamine
WT: Wild type.
