# Supplementary material for: Molecular surveillance of pvdhfr, pvdhps, and pvmdr-1 mutations in Plasmodium vivax isolates from Yunnan and Anhui provinces of China
Source: Malar J. 2014 Sep 2;13:346. doi: 10.1186/1475-2875-13-346 (PMC4161776; doi:10.1186/1475-2875-13-346)
Supplement: Supplementary file 1 — Additional file 1: Frequency distribution of SNPs in combination of pvdhfr , pvdhps , and pvmdr-1 haplotypes associated with SP and CQ in P. vivax isolates. Description: The data provided frequency distribution of SNPs in combination of pvdhfr, pvdhps, and pvmdr-1 haplotypes. (DOCX 34 KB) [file 12936_2014_3378_MOESM1_ESM.docx]

**Additional file 1**

**File format**: DOC

**Title: Frequency distribution of SNPs in combination of *pvdhfr*, *pvdhps*, and *pvmdr-1* haplotypes associated with SP and CQ in *P. vivax* isolates.**

**Description:** The data provided frequency distribution of SNPs in combination of *pvdhfr*, *pvdhps*, and *pvmdr-1* haplotypes.

Table 1. **Frequency distribution of SNPs in combination of *pvdhfr*, *pvdhps*, and *pvmdr-1* haplotypes associated with SP and CQ in *P. vivax* isolates ^a^**

|  | ***pvdhfr*** | | | | |  | ***pvdhps*** | | | |  | ***pvmdr-1*** | |  | **Number of isolates (%)** | | |
| --- | --- | --- | --- | --- | --- | --- | --- | --- | --- | --- | --- | --- | --- | --- | --- | --- | --- |
| Haplotype | **F57** | **S58** | **T61** | **H99** | **S117** |  | **S382** | **A383** | **K512** | **A553** |  | **Y976** | **F1076** |  | **Subtropical zone, *n* = 53** | **Temperate zone, *n* = 61** | **Total**  ***n* = 114** |
|  |  |  |  |  |  |  |  |  |  |  |  |  |  |  |  |  |  |
| 1 | F | S | T | H | S |  | S | A | K | A |  | Y | F |  | 1 (1.9) | 0 (0) | 1 (0.9) |
| 2 | F | S | T | H | S |  | S | A | K | A |  | Y | **L** |  | 4 (7.5) | 11 (18.0) | 15 (13.2) |
| 3 | F | S | T | H | S |  | S | **G** | K | **G** |  | Y | F |  | 1 (1.9) | 0 (0) | 1 (0.9) |
| 4 | F | S | T | H | S |  | S | **G** | **E** | **G** |  | Y | **L** |  | 1 (1.9) | 0 (0) | 1 (0.9) |
| 5 | F | S | T | H | S |  | **A** | **G** | K | A |  | Y | F |  | 1 (1.9) | 0 (0) | 1 (0.9) |
| 6 | F | S | T | H | **N** |  | S | A | K | A |  | Y | F |  | 1 (1.9) | 0 (0) | 1 (0.9) |
| 7 | F | S | T | H | **N** |  | S | A | K | A |  | Y | **L** |  | 0 (0) | 26 (42.6) | 26 (22.8) |
| 8 | F | S | T | H | **N** |  | **A** | **G** | K | **G** |  | Y | F |  | 1 (1.9) | 0 (0) | 1 (0.9) |
| 9 | F | S | T | **S** | S |  | S | A | K | A |  | Y | F |  | 1 (1.9) | 0 (0) | 1 (0.9) |
| 10 | F | S | T | **S** | S |  | S | A | K | A |  | Y | **L** |  | 2 (3.8) | 15 (24.6) | 17 (14.9) |
| 11 | F | S | T | **S** | S |  | S | **G** | K | A |  | Y | F |  | 1 (1.9) | 0 (0) | 1 (0.9) |
| 12 | F | S | T | **S** | S |  | **A** | **G** | K | A |  | Y | **L** |  | 2 (3.8) | 0 (0) | 2 (1.8) |
| 13 | F | S | T | **S** | **N** |  | S | A | K | A |  | Y | **L** |  | 0 (0) | 9 (14.8) | 9 (7.9) |
| 14 | F | S | T | **S** | **N** |  | S | **G** | K | A |  | Y | F |  | 1 (1.9) | 0 (0) | 1 (0.9) |
| 15 | F | S | T | **R** | **N** |  | S | **G** | K | A |  | Y | F |  | 3 (5.7) | 0 (0) | 3 (2.6) |
| 16 | F | **R** | T | H | **N** |  | S | **G** | K | A |  | **F** | **L** |  | 1 (1.9) | 0 (0) | 1 (0.9) |
| 17 | **I** | **R** | **M** | H | **T** |  | S | **G** | K | A |  | Y | F |  | 1 (1.9) | 0 (0) | 1 (0.9) |
| 18 | **I** | **R** | **M** | H | **T** |  | S | **G** | K | **G** |  | Y | F |  | 2 (3.8) | 0 (  0  ) | 2 (1.8) |
| 19 | **I** | **R** | **M** | H | **T** |  | **A** | **G** | K | A |  | **F** | **L** |  | 1 (1.9) | 0 (  0  ) | 1 (0.9) |
| 20 | **L** | **R** | **M** | H | **T** |  | S | A | K | A |  | Y | F |  | 2 (3.8) | 0 (  0  ) | 2 (1.8) |
| 21 | **L** | **R** | **M** | H | **T** |  | S | **G** | K | A |  | Y | F |  | 6 (11.3) | 0 (  0  ) | 6 (5.2) |
| 22 | **L** | **R** | **M** | H | **T** |  | S | **G** | K | **G** |  | Y | F |  | 7 (13.2) | 0 (  0  ) | 7 (6.1) |
| 23 | **L** | **R** | **M** | H | **T** |  | S | **G** | K | **G** |  | Y | **L** |  | 1 (1.9) | 0 (  0  ) | 1 (0.9) |
| 24 | **L** | **R** | **M** | H | **T** |  | **A** | **G** | K | A |  | Y | F |  | 4 (7.6) | 0 (  0  ) | 4 (3.5) |
| 25 | **L** | **R** | **M** | H | **T** |  | **A** | **G** | K | A |  | Y | **L** |  | 3 (5.7) | 0 (  0  ) | 3 (2.6) |
| 26 | **L** | **R** | **M** | H | **T** |  | **A** | **G** | K | A |  | **F** | **L** |  | 3 (5.7) | 0 (  0  ) | 3 (2.6) |
| 27 | **L** | **R** | **M** | H | **T** |  | **A** | **G** | K | **G** |  | Y | F |  | 1 (1.9) | 0 (  0  ) | 1 (0.9) |
| 28 | **L** | **R** | **M** | H | **T** |  | **A** | **G** | K | **G** |  | Y | **L** |  | 1 (1.9) | 0 (  0  ) | 1 (0.9) |

^a^ In isolates from subtropical (Yunnan province) and temperate (Anhui province) zones of China.
